# Supplementary material for: Precise Detection and Visualization of Nanoscale Temporal Confinement in Single-Molecule Tracking Analysis
Source: Membranes (Basel). 2022 Jun 24;12(7):650. doi: 10.3390/membranes12070650 (PMC9320997; doi:10.3390/membranes12070650)
Supplement: Supplementary file 1 [file membranes-12-00650-s001.zip › membranes-1752671-supplementary.pdf]

membranes

Supplementary Materials

Precise Detection and Visualization of Nanoscale Temporal Confinement in Single-Molecule Tracking Analysis

Manon Westra and Harold D. MacGillavry *

Cell Biology, Neurobiology and Biophysics, Department of Biology, Faculty of Science, Utrecht University, 3584 CH Utrecht, The Netherlands; m.westra@uu.nl
* Correspondence: h.d.macgillavry@uu.nl

Citation: Westra, M.; MacGillavry, H.D. Precise Detection and Visualization of Nanoscale Temporal Confinement in Single-Molecule Tracking Analysis. *Membranes* **2022**, *12*, 650. https://doi.org/10.3390/membranes12070650

Academic Editors: Shiro Suetsugu and Yosuke Senju

Received: 17 May 2022
Accepted: 22 June 2022
Published: 24 June 2022

Publisher's Note: MDPI stays neutral with regard to jurisdictional claims in published maps and institutional affiliations.

Copyright: © 2022 by the authors. Submitted for possible open access publication under the terms and conditions of the Creative Commons Attribution (CC BY) license (https://creativecommons.org/licenses/by/4.0/).

Table S1. Confinement parameters and their effect on the power of detection.

Parameter	Definition	Value in this study	Effect on confinement detection
Lc	Critical *L*; threshold for confinement detection	α * average *L*	
Lcm	Minimum critical *L*; L below this value will be random diffusion	5.5	Lower *Lcm* will increase false positives, higher *Lcm* will decrease true positives
Tc	Critical time; minimal time period *L* should be above *Lc*	0.2 s	Lower *Tc* will increase false positives, higher *Tc* will decrease true positives
Sm	Maximum segment length for confinement detection	15 frames	Larger *Sm* will increase power of detection, but reduces the temporal resolution
Smin	Minimum segment length for confinement detection	4 frames	No large effect on confinement detection
α	Average *L* of a trajectory is multiplied by this factor in the range of 0-1 resulting in *Lc*	0.5	Low value will lead to *Lc* close to *Lcm*, high value could increase power of detection when there are highly confined zones but could also merge multiple confinement zones

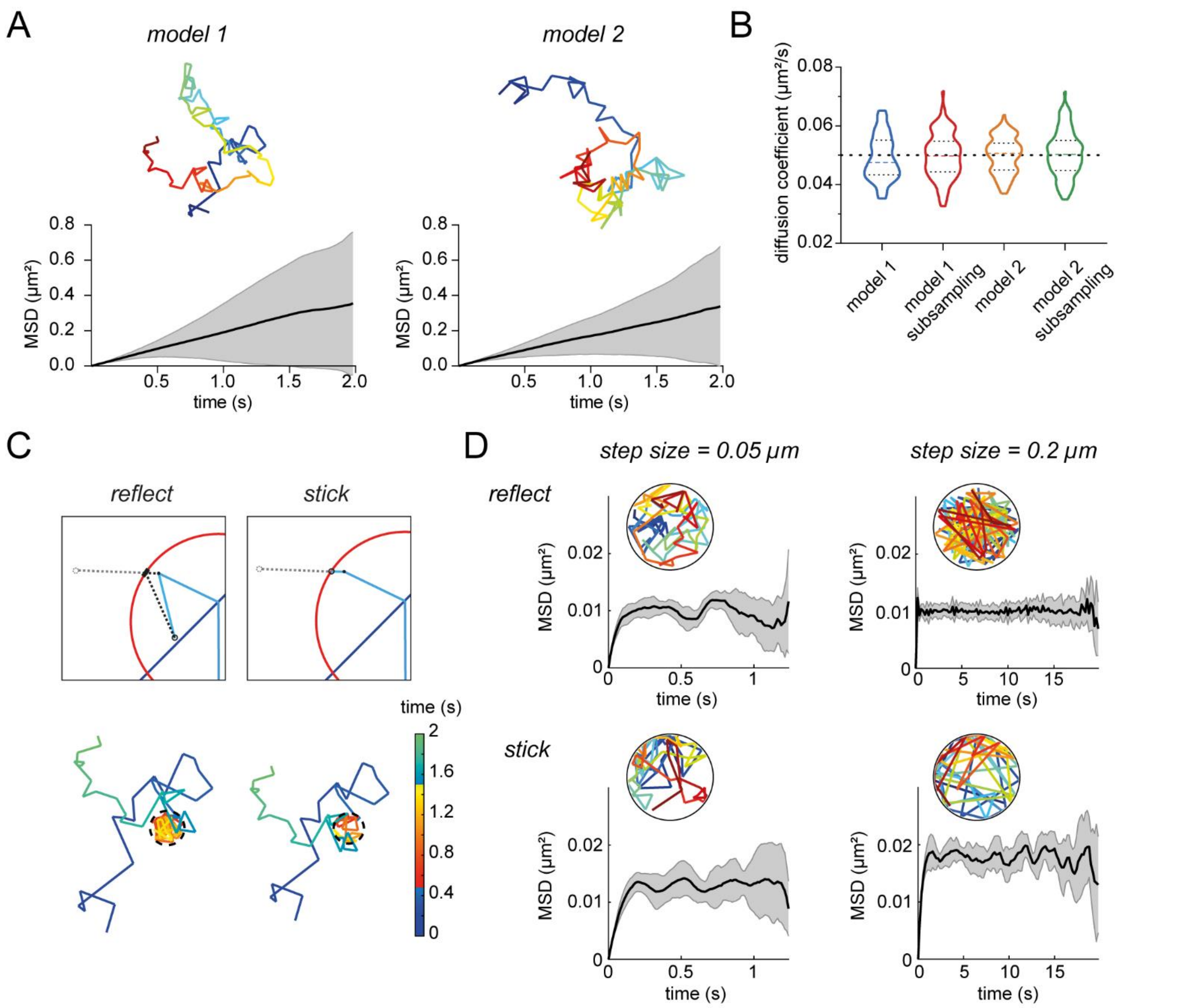

Figure S1. Random walk and confinement simulations. (**A**) Model 1: random walk simulation where every consecutive coordinate is drawn from a Gaussian distribution based on diffusion coefficient and time step (left). Model 2: random walk simulation where step length is the same for every step, but the direction of the step is random (right). MSD versus time lag curves of 100 random walks (bottom). (**B**) Distribution of diffusion coefficients for the different random walk simulation models and after applying subsampling: only including the coordinate after every 100 simulation steps. (**C**) Principle of the 'reflect' confinement model (left). Principle of the 'stick' confinement model (right). Example trajectory for both models color-coded for time (bottom). *Rconf* = 0.05 µm. (**D**) MSD curves for 10 completely confined tracks simulated with the 'reflect' model (top) and the 'stick' model. Comparing a step size of 0.05 µm (left) and 0.2 µm (right). Grey area represents the weighted standard deviation over all MSD curves. *Rconf* = 0.1 µm.

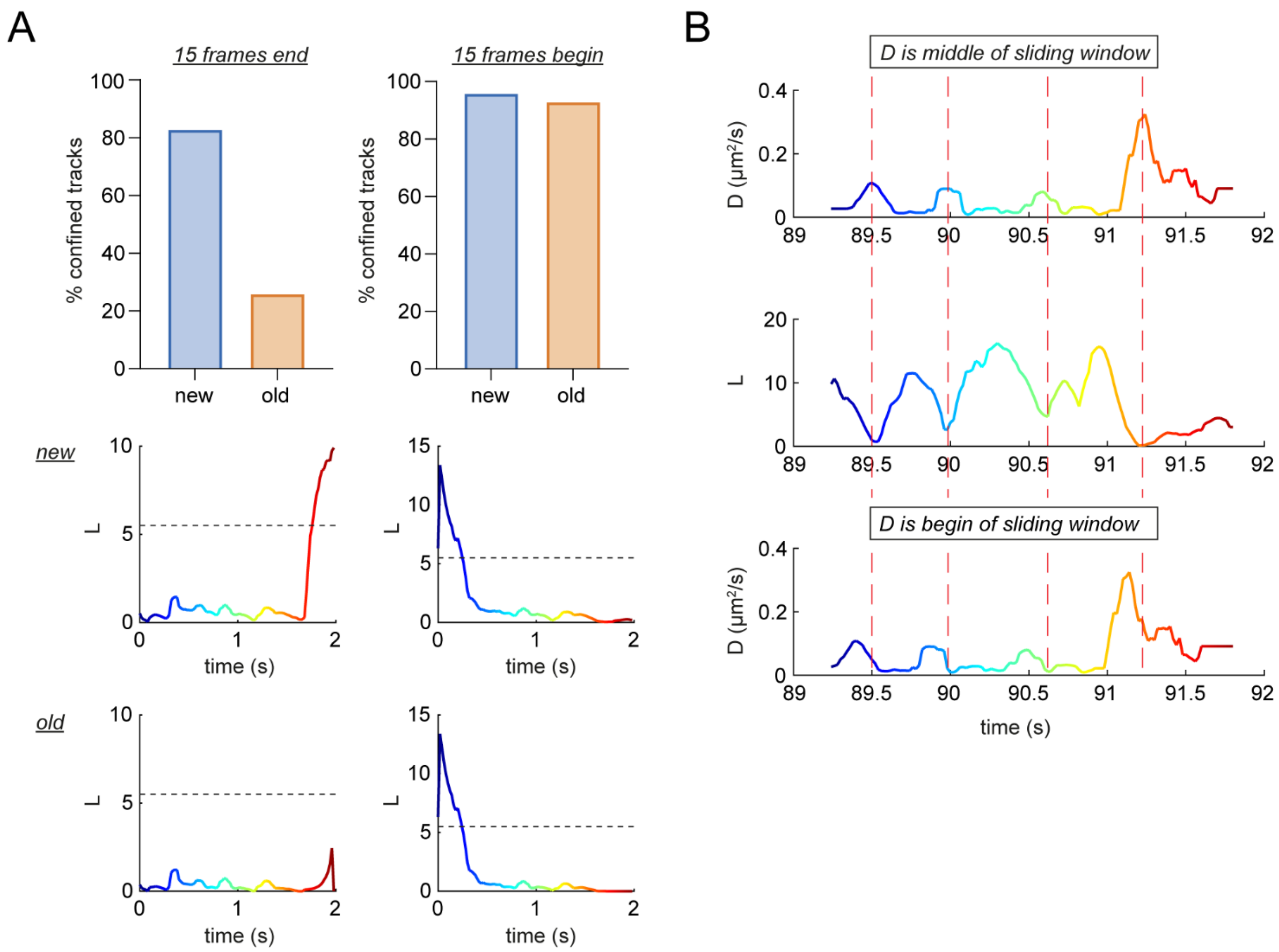

Figure S2. Improvements in the confinement analysis. (**A**) Including the last points of a trajectory in the confinement index averaging. 100 trajectories were simulated with the last 15 frames of 100 frames confined. Comparison of the analysis from [1] (old) and our updated analysis (new). Our analysis achieves better detection of confinement periods at the end of the trajectory (left). There is no large difference in the detection of confinement at the beginning of trajectories (right). 100 simulated trajectories with the first 15 frames of 100 frames confined. *Rconf* = 0.04 µm. Confinement index (L) over time is shown for example trajectories analyzed with the updated method (middle) and the method from [1] (bottom). (**B**) Use D as the middle of the sliding window instead of the beginning. Peaks in the diffusion coefficient over time are indicated with the dashed red lines and correspond with valleys in the confinement index over time graph, as expected. This correlation of peaks and valleys is not as prominent when D is used as the beginning of the sliding window (bottom).

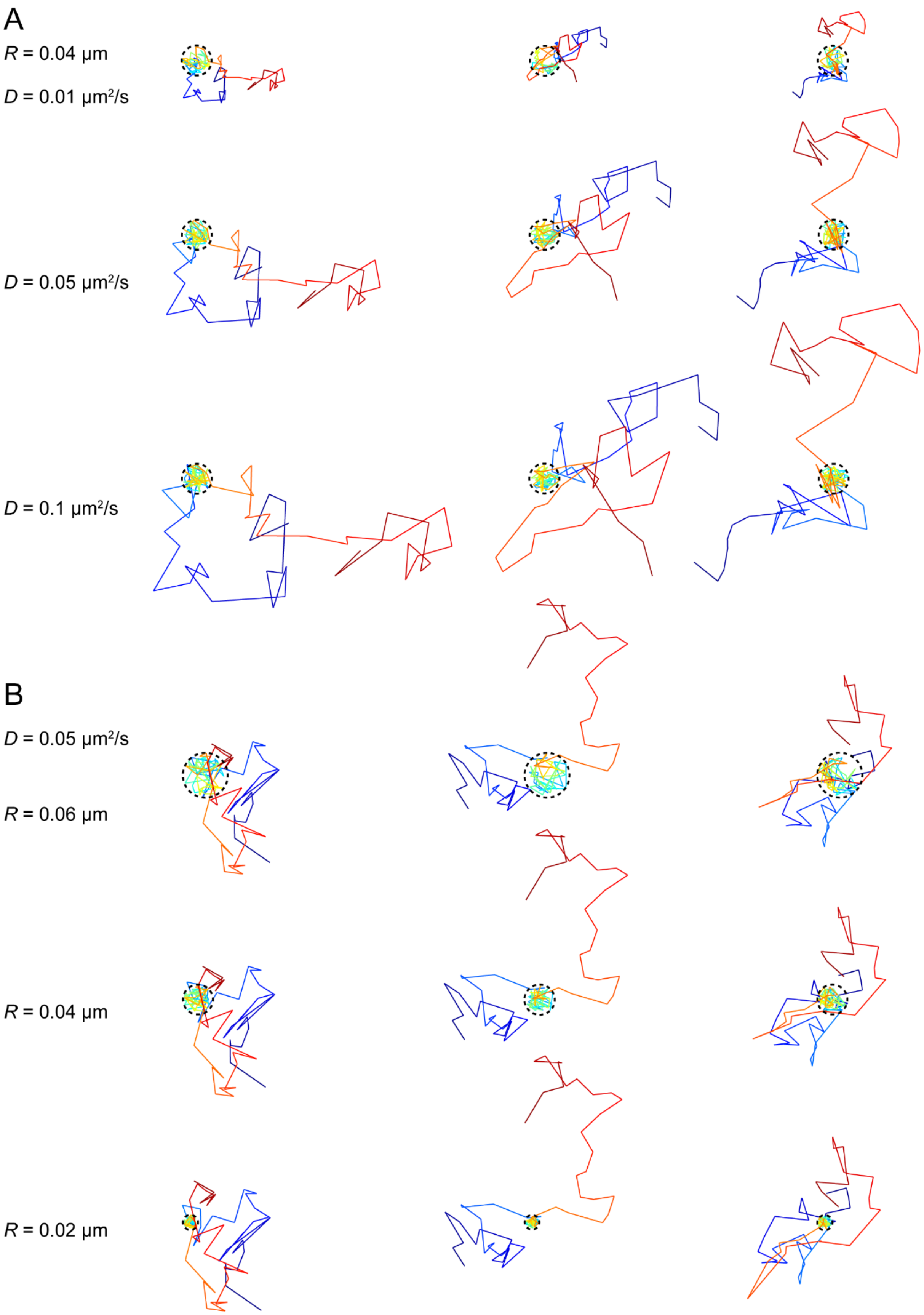

Figure S3. Raw simulated transient confined trajectories from Figure 1. (**A**) Three different simulated trajectories varied in the diffusion coefficient of the simulation. *Rconf* = 0.04 μm. (**B**) Three different simulated trajectories varied in their confinement radius size. D = 0.05 μm^2/s.

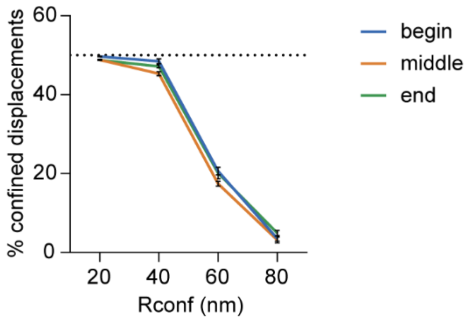

Figure S4. Effect of timing confinement on power of detection. Effect of the timing of simulated confinement periods and *Rconf* on the correct detection of that confinement. 50 frames confined of total 100 frames per simulated track. Five independent simulations of 100 trajectories per condition. Dotted line indicates correct confinement detection. Data are represented as means ± SD.

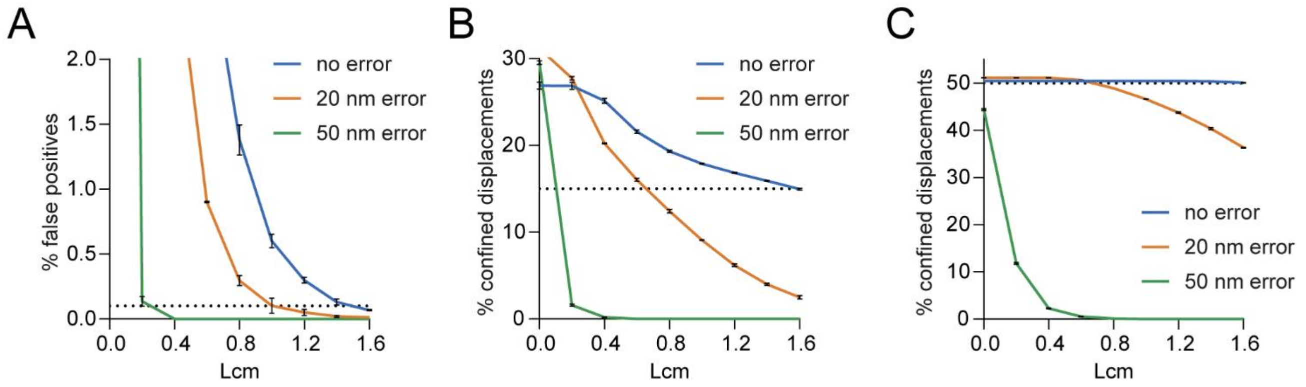

Figure S5. Effect of localization error on confinement detection using Dset. (**A**) Effect of localization error on the percentage of false positives detected in random walks using Dset instead of Dmax. 3 independent simulations of 1000 trajectories per condition. Data are represented as means ± SD. (**B**, **C**) Effect of localization error on the percentage of detected confined displacements in tracks simulated to be confined for 15 (**B**) or 50 (**C**) of the 100 frames. *Rconf* = 0.04 µm. 3 independent simulations of 1000 trajectories. Data are represented as means ± SD.

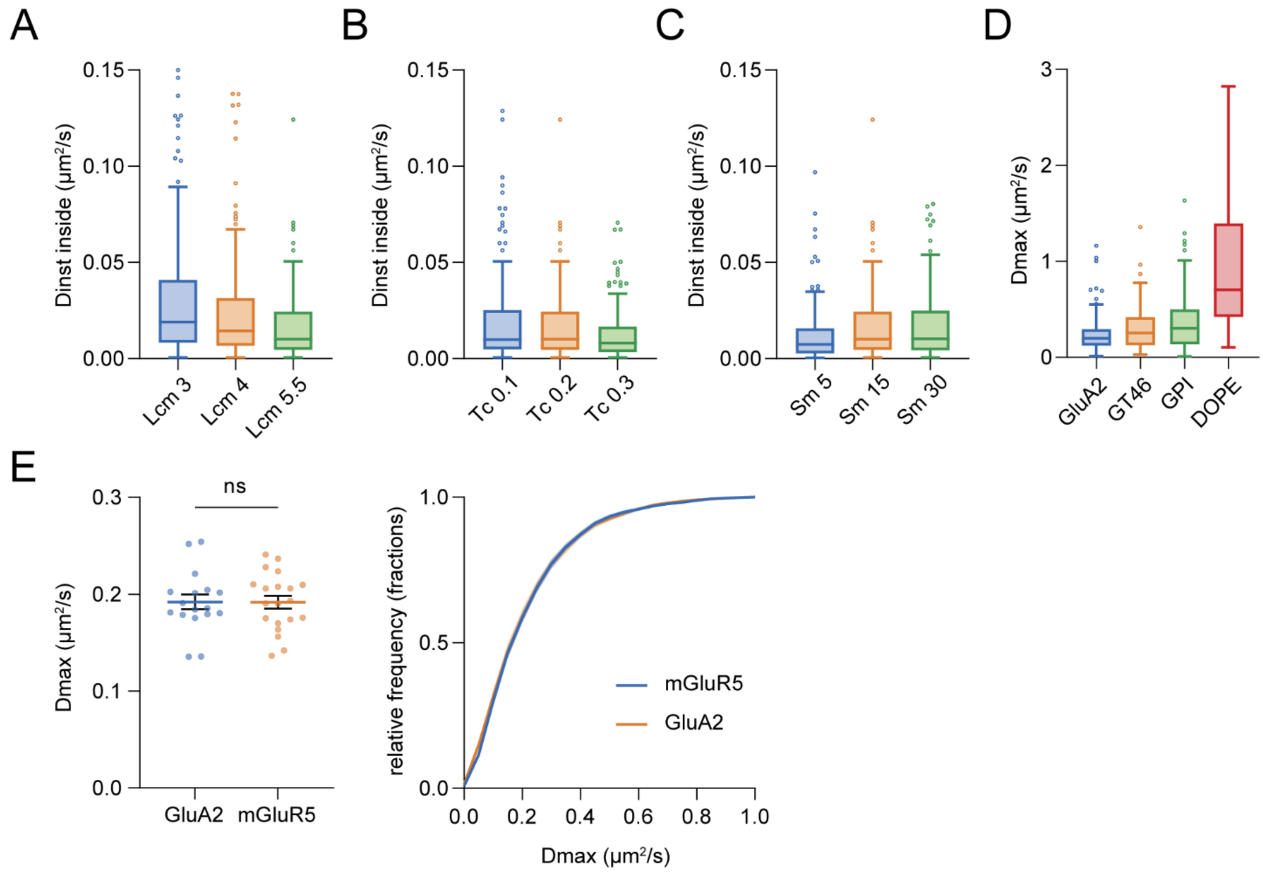

Figure S6. Confinement analysis on experimental trajectories. (**A**–**C**) Effect of user-defined parameters on the estimated diffusion coefficient inside confinement zones. (**A**) Vary critical *L*, *Lcm*. Number of trajectories with confinement: *Lcm* 3: 381, *Lcm* 4: 309, *Lcm* 5.5: 219. (**B**) Vary critical time, *Tc*. Number of trajectories with confinement: *Tc* 0.1: 311, *Tc* 0.2: 219, *Tc* 0.3: 139. (**C**) Vary maximum segment length, *Sm*. Number of trajectories with confinement: *Sm* 5: 181, *Sm* 15: 219, *Sm* 30: 177. (**D**) Estimated Dmax for membrane probes: GluA1, GT46, GPI and DOPE. (**E**) Average estimated Dmax for mGluR5 (n = 20) and GluA2 (n = 17; unpaired t-test) (left), and corresponding cumulative frequency distribution (right). Data are represented as means ± SEM.

References

1. Menchón, S.A.; Martín, M.G.; Dotti, C.G. APM_GUI: Analyzing particle movement on the cell membrane and determining confinement. *BMC Biophys.* **2012**, *5*, 4. https://doi.org/10.1186/2046-1682-5-4.
